# Supplementary material for: Long- and Short-Term Glucosphingosine (lyso-Gb1) Dynamics in Gaucher Patients Undergoing Enzyme Replacement Therapy
Source: Biomolecules. 2024 Jul 12;14(7):842. doi: 10.3390/biom14070842 (PMC11275231; doi:10.3390/biom14070842)
Supplement: Supplementary file 1 [file biomolecules-14-00842-s001.zip › biomolecules-3050115-supplementary/Suppl table S1.pdf]

Supplementary Table S1. Long term observation of lyso-Gb1 level [ng/ml] for patients who had at least six out of eight measurements taken (n.a. – non analyzed).

ID	GD1/3	Lyso-gb1 level [ng/ml]							
		2016	2017	2018	2019	2020	2021	2022	2023
2	1	200,2	284,2	206,54	195,2	188,6	154,6	157	313,4
4	1	54,4	98,9	130,44	112,1	94,4	61,9	39,8	43,4
7	1	24,7	68,4	67,62	40,7	81,8	62,2	42	78,5
8	1	55,2	68,8	70,83	54	128,9	309,1	n.a.	n.a.
9	1	26,1	41,5	36,42	28,6	38,7	37,4	44,1	53,3
10	1	147	164,9	112,13	84,4	161,7	82,5	68,1	68,9
11	1	255,6	92,2	32,49	22,4	31,9	23,8	32,8	44,9
12	1	52,6	58,2	50,217	37,4	73,3	61	63,8	99,9
13	1	112,3	133,7	n.a.	128,1	153,2	108,3	124,7	148,4
14	1	69,9	70,1	75,56	388,7	50,7	75,6	34,1	n.a.
15	1	386,2	380	379,57	244,3	216,8	n.a.	267	n.a.
16	1	35,6	42,1	42,5	31,9	48,1	n.a.	42,5	53,9
20	1	72,6	56,9	55,7	51,3	51,3	n.a.	n.a.	n.a.
21	1	381,2	557	621,94	364	505,4	n.a.	480,7	452,1
23	1	49,4	64,4	72,95	73,6	85	88,4	78,5	110,2
25	1	529	1151,4	1600,22	762,9	242,3	132	89	124,5
26	1	44,4	64,4	61,3	40,9	40,9	n.a.	57	82,7
28	1	72,3	82,2	79,54	71,5	72,2	82,4	55	32,1
29	1	42,5	74,4	53,2	40,6	57,3	n.a.	n.a.	33,8
30	1	246,9	250,6	283,6	195,5	n.a.	227,9	212,7	286,1
31	1	493,4	640,4	545,4	447,3	447,3	n.a.	212,6	28,7
33	1	125,3	146,9	190,81	116,4	221,6	178,3	183,4	262,8
34	1	n.a.	142,9	161,69	59,8	114,4	72,3	78,6	100,6
35	1	55,6	55	70,9	48,6	n.a.	n.a.	50,7	68
36	1	25,1	29,1	37,07	26,8	36,4	31,4	30,3	30,6
37	1	33,9	34,4	36,18	31,3	38,8	n.a.	38,5	n.a.
38	1	169,6	201,6	144,31	73	106	50	42,1	n.a.
39	1	29,9	37,9	44,94	39,5	51,2	51,3	54,2	58,3
40	1	n.a.	n.a.	94,55	74	94,6	n.a.	n.a.	n.a.
43	1	40,2	41,1	50,32	41,7	41,7	74,3	64,4	76,2
44	1	33,9	38,9	40,08	34,4	44	36,9	43	50,9
45	1	119,6	199,2	179,16	n.a.	n.a.	577,2	12,2	535
46	1	76,6	41,8	43,81	49,7	65,4	47,3	n.a.	81,3
47	1	47,5	57,1	59,77	42,5	79,1	n.a.	102,3	103,5
57	3	58,6	76,3	79,37	62,4	68,7	78,5	115,7	103,8
58	3	84,3	113,8	111,7	71,9	88,1	n.a.	102,7	115,9
59	3	77,4	79,8	77,9	64,3	n.a.	n.a.	62,1	76,5
62	3	651,5	947,8	947,8	522,7	965,1	836	1242,9	n.a.

**63**	3	35,8	48,7	63,13	31,8	n.a.	64,2	59,4	87,6
**64**	3	26,1	27,3	35,72	24,7	44,3	41,3	52,7	42,8
**66**	3	121,7	120,7	160,43	98,4	133,6	118.1	117,8	123,1
**67**	3	57,7	63,8	72,61	65	n.a.	73,7	59,3	75,5
**68**	3	897	749,2	751,76	693,8	856,3	724,8	858	761,8
**69**	3	40,8	81,5	79,12	64,8	80,7	85	n.a.	108,2
**70**	3	58	70,2	61,95	42,7	67,2	64.6	57,1	78,7
**72**	3	43	62	60,91	52,6	70,6	68,8	49,6	98,6
**73**	3	184,6	166.5	220,13	207	311,9	204	256,9	236,5
**74**	3	85,6	133,2	103,52	80,7	104,2	128,7	123,9	196,3
**75**	3	234,1	245,9	470,82	131,1	157,5	333,3	194,3	148,9
