# Supplementary material for: Long- and Short-Term Glucosphingosine (lyso-Gb1) Dynamics in Gaucher Patients Undergoing Enzyme Replacement Therapy
Source: Biomolecules. 2024 Jul 12;14(7):842. doi: 10.3390/biom14070842 (PMC11275231; doi:10.3390/biom14070842)
Supplement: Supplementary file 1 [file biomolecules-14-00842-s001.zip › biomolecules-3050115-supplementary/Suppl table S2.pdf]

Supplementary Table S2 Lyso-gb1 in years in GD1 and GD3 cohorts

	Mean lyso-gb1 concentration [ng/ml/patient]							
**Year**	**2016**	**2017**	**2018**	**2019**	**2020**	**2021**	**2022**	**2023**
**GD1 with all results (n = 16)**	110	159	178	110	100	83	77	100
**GD3 with all results (n = 9)**	190	83	216	155	202	196	210	199
