# Supplementary figures and images for: Long- and Short-Term Glucosphingosine (lyso-Gb1) Dynamics in Gaucher Patients Undergoing Enzyme Replacement Therapy
Source: Biomolecules. 2024 Jul 12;14(7):842. doi: 10.3390/biom14070842 (PMC11275231; doi:10.3390/biom14070842)

**Supplementary Figure S1**  
**Flow chart of the patients included in the study**

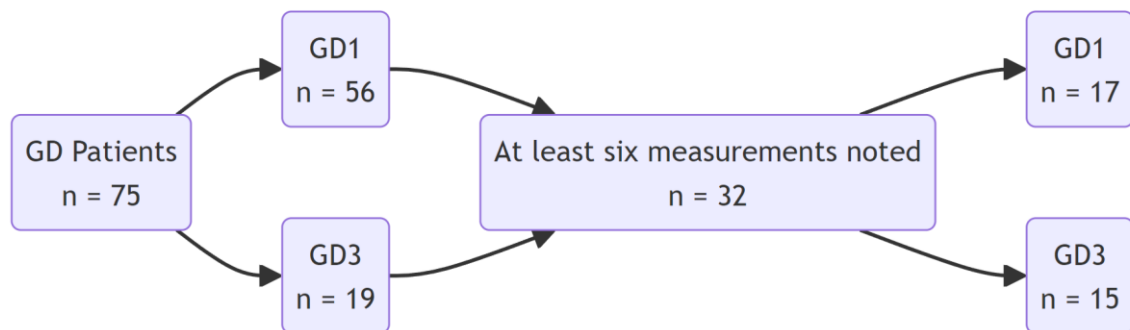

Supplement: Supplementary file 1 [file biomolecules-14-00842-s001.zip › biomolecules-3050115-supplementary/Supplementary Figure 1_corr.pdf]
